# A Comparison of Functional Outcome in Patients Sustaining Major Trauma: A Multicentre, Prospective, International Study

**DOI:** 10.1371/journal.pone.0103396

**Published:** 2014-08-26

**Authors:** Timothy H. Rainer, Hiu Hung Yeung, Belinda J. Gabbe, Kai Y. Yuen, Hiu F. Ho, Chak W. Kam, Annice Chang, Wai S. Poon, Peter A. Cameron, Colin A. Graham

**Affiliations:** 1 Accident and Emergency Medicine Academic Unit, Chinese University of Hong Kong, Shatin, New Territories, Hong Kong SAR, China; 2 Trauma & Emergency Centre, Prince of Wales Hospital, Shatin, New Territories, Hong Kong SAR, China; 3 Department of Epidemiology and Preventive Medicine, Monash University, Melbourne, Victoria, Australia; 4 College of Medicine, Swansea University, West Glamorgan, Wales, United Kingdom; 5 Accident and Emergency Department, Queen Elizabeth Hospital, Yau Ma Tei, Kowloon, Hong Kong SAR, China; 6 Accident and Emergency Department, Tuen Mun Hospital, Tuen Mun, New Territories, Hong Kong SAR, China; 7 Division of Neurosurgery, Department of Surgery, Chinese University of Hong Kong, Shatin, New Territories, Hong Kong SAR, China; 8 Emergency and Trauma Centre, Alfred Hospital, Melbourne, Victoria, Australia; Cardiff University, United Kingdom

## Abstract

**Objectives:**

To compare 6 month and 12 month health status and functional outcomes between regional major trauma registries in Hong Kong and Victoria, Australia.

**Summary Background Data:**

Multicentres from trauma registries in Hong Kong and the Victorian State Trauma Registry (VSTR).

**Methods:**

Multicentre, prospective cohort study. Major trauma patients and aged ≥18 years were included. The main outcome measures were Extended Glasgow Outcome Scale (GOSE) functional outcome and risk-adjusted Short-Form 12 (SF-12) health status at 6 and 12 months after injury.

**Results:**

261 cases from Hong Kong and 1955 cases from VSTR were included. Adjusting for age, sex, ISS, comorbid status, injury mechanism and GCS group, the odds of a better functional outcome for Hong Kong patients relative to Victorian patients at six months was 0.88 (95% CI: 0.66, 1.17), and at 12 months was 0.83 (95% CI: 0.60, 1.12). Adjusting for age, gender, ISS, GCS, injury mechanism and comorbid status, Hong Kong patients demonstrated comparable mean PCS-12 scores at 6-months (adjusted mean difference: 1.2, 95% CI: −1.2, 3.6) and 12-months (adjusted mean difference: −0.4, 95% CI: −3.2, 2.4) compared to Victorian patients. Keeping age, gender, ISS, GCS, injury mechanism and comorbid status, there was no difference in the MCS-12 scores of Hong Kong patients compared to Victorian patients at 6-months (adjusted mean difference: 0.4, 95% CI: −2.1, 2.8) or 12-months (adjusted mean difference: 1.8, 95% CI: −0.8, 4.5).

**Conclusion:**

The unadjusted analyses showed better outcomes for Victorian cases compared to Hong Kong but after adjusting for key confounders, there was no difference in 6-month or 12-month functional outcomes between the jurisdictions.

## Introduction

In order to improve the quality of survival of trauma patients, there is a worldwide impetus to develop and improve trauma systems [1], [2]. In 2003, the Hospital Authority in Hong Kong designated five hospitals as trauma centres [3] and since then there has been a gradual improvement in trauma survival [4]. However Hong Kong still lags behind Australia in trauma system development and survival from major injury [5].

It is important to look beyond mere survival, and to assess both mental and physical aspects of functional outcome [6]. Survivors of trauma often experience late sequelae that have a major impact on almost all aspects of their everyday life [7]–[9]. Patient-centred, health-related outcomes are increasingly recognized as an important benchmark of the quality of care received. Meaningful comparisons between different centres enable healthcare providers to assess how well they are doing and where they might target future development. Comparable registries have been developed in Australia and Hong Kong [10]–[12]. In the last five years, there have been published reports from Australia [13]–[20] and Hong Kong [6] on functional outcome and the impact of introducing an inclusive trauma system on reducing road transport-related serious injury [21].

There is little information about the recovery of survivors of moderate and major trauma in Hong Kong. We hypothesise that there is no difference in post-trauma functional health status in major trauma patients at 6 and 12 months after injury between Hong Kong and Victoria but in view of our previous study which showed a better survival outcome in Victoria [5], we expect to reject the hypothesis. Our ability to exclude a type I or type II error was unclear as there was no previous data on which to estimate an appropriate sample size. Therefore we have conducted an exploratory study with a view to addressing the hypothesis but also shedding light on research of this nature.

The aim of this prospective cohort study was to compare patients' functional health status after major trauma between Hong Kong and Victoria, Australia. Specifically we aimed to compare quality of life and functional outcome using the Short-Form 12 (SF-12) health status tool, and the extended Glasgow Outcome Scale (GOSE), respectively.

## Methods

### Study design

Ethical approval was obtained from the joint CUHK-NTEC Clinical Research Ethics Committee in Hong Kong, and from the Standing Committee on Ethics in Research Involving Humans at Monash University and by all participating institutions in Victoria, Australia to conduct a prospective cohort study in patients with moderate and major trauma. In Hong Kong, written informed consent was given by all participants (or next of kin) for their clinical records to be used in this study. Patient records/information were also anonymized and de-identified prior to analysis in all cases. In Victoria, The registry uses an opt-off consent process where all eligible cases are included on the registry, and patients (or their next of kin) are provided with a letter and a brochure stating the aims of the registry, the data collected, and that patients will be followed-up. The brochure provides the details for how to opt-off and the opt-off rate for the registry is less than 1%. At the follow-up interview, verbal consent to complete the interview is obtained. An opt-off consent is used due to the impracticability of informed consent, and the potential for selection bias, in the registry setting. The registry protocol, including the described consent process, has been approved by the Human Research Ethics Committee of each participating hospital and Monash University. The exclusion rate in the Victorian State Trauma Registry (VSTR) is <0.5%.

Data were extracted from (VSTR), in Australia, and from the Prince of Wales Hospital (PWH) Trauma Registry, New Territories East Cluster, from the Queen Elizabeth Hospital (QEH) Trauma Registry, Kowloon East Cluster, and the Tuen Mun Hospital Trauma Registry in the Western New Territories of Hong Kong. Patients were recruited between 1^st^ January 2010 and 15^th^ September 2010.

### Hong Kong

In Hong Kong, the population is 7 million of which 95% are Chinese. The PWH, QEH and TMH trauma registries are hospital based registries, which cover the New Territories and parts of Kowloon. The population served by the three trauma centres in Hong Kong approximate to less than 5 million over less than 1,000 km^2^. The inclusion criteria for PWH, QEH and TMH include trauma deaths, patients triaged as ‘critical’ or ‘emergency’ in the Emergency Department (triage categories 1 and 2), all ICU admissions, and major trauma patients transferred from another acute hospital.

### Victoria

In Australia, the population of the State of Victoria is approximately 5.5 million people, accounting for 24% of the Australian population. Victoria is a state in southern Australia and the VSTR is a state-wide population-based trauma registry which was developed in 2001, and is based at Monash University, Melbourne. Two thirds of the Victorian population live in metropolitan Melbourne. The Victoria storm recruits patients from a populated area of 5.4 million, over more than 220,000 km^2^ and 138 trauma receiving hospitals. Definitive care of major trauma patients is centralised to one pediatric and two adult major trauma centres, which capture more than 80% of major trauma patients. In an integrated trauma system with metropolitan and regional services, 138 health service facilities contribute data to the VSTR. The registry enables tracking of cases across the system by collecting identifiable information.

### Patients, inclusion and exclusion criteria

All adult patients aged ≥18 years with major trauma (defined as an ISS≥16) who were entered into the trauma registries of Hong Kong and VSTR between 1 January 2010 and 15^th^ September 30 June and who survived to hospital discharge were included in the study.

### Instruments

The evaluation of the physical and mental health status of trauma patients (objective 1) utilised the physical component summary (PCS-12) score and mental component summary (MCS-12) scores respectively of the generic 12-item Short-Form Health Survey (SF-12) [22]–[23]. The evaluation of functional outcome was assessed at baseline, one month, six months and 12 months using the extended Glasgow Outcome Scale (GOSE) [24]–[25]. The SF is well-validated, reliable and sensitive to change and has been extensively used to assess and follow up trauma patients [26]–[27]. There are UK, US, Australian, Chinese and Hong Kong specific ‘population norms’ for the major subdivisions and subscales of the SF which allow meaningful comparisons of health status between the group of interest and the general population [28]–[29]. Population norms are defined as the mean for that population. Standard deviations are not reported in this context. The population norms for the PCS and MCS in HK are 52.83 and 47.18 respectively, for the US are 50.12 and 50.04 respectively, and for Australia are 49.79 and 50.01 respectively. For comparison between HK and VSTR, the SF-12 was used. US weights were used for both the Hong Kong and Australian data.

### Measurements and Data Collection

Demographic data including age, sex, comorbidity, mechanism of injury, Injury Severity Score (ISS) [30]–[31]. Revised Trauma Score (RTS) [32], probability of survival (Ps) [33], Glasgow Coma Scale (GCS), hospital and ICU length of stay (LOS) and contact information were extracted from the trauma registry and patients' records.

Cases were considered to have a comorbid condition if they had any one of the Charlson Comorbidity Index conditions: Myocardial infarction (history, not ECG changes only); Congestive heart failure; Peripheral disease (includes aortic aneurysm > = 6 cm; Cerebrovascular disease: CVA with mild or no residua or TIA; Dementia; Chronic pulmonary disease; Connective tissue disease; Peptic ulcer disease; Mild liver disease (without portal hypertension, includes chronic hepatitis); Diabetes without end-organ damage (excludes diet-controlled alone); Hemiplegia; Moderate or severe renal disease Diabetes with end-organ damage (retinopathy, neuropathy,nephropathy, or brittle diabetes); Tumor without metastasis (exclude if >5 y from diagnosis); Leukemia(acute or chronic); Lymphoma; Moderate or severe liver disease; Metastatic solid tumor.

Probability of survival is calculated using the TRISS (Trauma Score and the Injury Severity Score) methods. The TRISS method is a way of standardising the evaluation of post-trauma mortality. Anatomical (ISS), physiological (RTS), age and calculated weights are used to quantify probability of survival as related to severity of injury. Thus, TRISS offers a means of case identification for quality assurance review on a local basis, as well as a means of comparison of outcome for different populations of trauma patients.'

Injured patients were classified according to whether injuries were isolated or multiple, and according to specific body regions – head and neck injury, chest injury, abdominal injury and extremity injury. Isolated injury was defined as a single AIS≥3. Multiple injury was defined as two or more regions with AIS≥3.

### Outcomes

The primary outcome was GOSE assessed at 6 months and 12 months. The secondary outcome was post-injury SF-12 score assessed at 6 months and 12 months. A responder was defined as a person who was successfully followed up – i.e. had a valid GOSE score. Non-responders were patients lost to follow-up at both time points.

### Statistical analyses

Summary statistics were used to describe the characteristics of major trauma patients from the Hong Kong and Victorian settings. Chi-square analysis was used to compare the trauma setting, and follow-up status, for categorical variables, while independent t-tests or Mann-Whitney U-tests were used for comparing data from continuous variables, depending on the distribution of the data.

The key outcomes of interest were the GOSE score at 6 and 12-months post-injury. The two lowest levels of the GOSE (death and vegetative state) were combined due to small numbers in the vegetative state category. The GOSE was then analysed with an ordered logistic regression model where GOSE was the dependent variable. The odds ratios (OR) and 95% confidence intervals (95% CI) estimated by this method compared the cumulative odds of belonging to a certain GOSE category or higher between groups of patients defined by the explanatory variables included in the model. The covariates used in the multivariate ordinal logistic regression were the setting (Hong Kong or Victoria), age, sex, ISS, GCS group (3–8, 9–12, or 13–15), mechanism of injury and comorbid status (healthy or pre-existing condition). Linear regression was used for analysis of SF-12 outcomes. All analyses were performed using Stata Version 11.2 (StataCorp, College Station, Texas, USA).

## Results

### Study population characteristics

In the VSTR, there were 1955 cases, 203 (10.4%) in-hospital deaths, leaving 1752 survivors to discharge. The exclusion rate in VSTR is <0.5%. In Hong Kong, during the study period, 593 potential cases were admitted to hospital of whom 332 were excluded (ISS<16, n = 139; patients died over 48 hours after injury but before assessment and consent were possible, n = 20; patients arrived out of office hours e.g. weekends or public holidays, and discharged before research assessment and consent were possible, n = 57; consent not possible because the patient was incapacitated by injury or prolonged procedures and relatives were not available to give consent, N = 114) leaving 261 cases for evaluation, 36 (13.9%) in-hospital deaths and 225 survivors to discharge. Table 1 shows the characteristics of major trauma patients from Victoria and Hong Kong.

**Table 1:** Characteristics of major trauma survivors to hospital discharge in Hong Kong and Victoria.

Descriptor		Victoria (n = 1955)	Hong Kong (n = 261)	p-value
Age	Mean (SD)	52.0 (22.0)	53.8 (20.3)	0.219
Sex	n (%)			
	Male	1378 (70.5)	187 (71.7)	0.699
	Female	577 (29.5)	74 (28.3)	
Comorbid status	n (%)			
	Healthy	1158 (59.2)	132 (52.0)	0.026
	Pre-existing condition	797 (40.8)	122 (48.0)	
Trauma type	n (%)			
	Blunt	1850 (94.6)	250 (95.8)	0.111
	Penetrating	75 (3.7)	4 (1.5)	
	Burn	33 (1.7)	7 (2.7)	
Mechanism	n (%)			
	Fall	719 (36.8)	144 (55.2)	<0.001
	Motor vehicle	428 (21.9)	23 (8.8)	
	Pedestrian	132 (6.8)	36 (13.8)	
	Motorcycle	225 (11.5)	23 (8.8)	
	Pedal cyclist	106 (5.4)	8 (3.1)	
	Other*	345 (17.6)	38 (14.6)	
ISS	Median (IQR)	18 (16–25)	24 (17–26)	<0.0001
GCS	n (%)			
	13–15	1532 (81.8)	192 (73.6)	0.004
	9–12	161 (8.6)	29 (11.1)	
	3–8	179 (9.6)	40 (15.3)	
ICU stay	n (%)			
	Yes	764 (39.2)	125 (47.9)	0.007
	No	1186 (60.8)	136 (52.1)	
Hospital length of stay	Median (IQR) days	7.8 (3.9–15.0)	12.2 (5.6–25.9)	<0.0001
In-hospital mortality	n (%)			
	No	1752 (89.6)	223 (86.1)	0.087
	Yes	203 (10.4)	36 (13.9)	

* Other causes of injury include: fire, flames, smoke and scalds, horse and other animal related injuries, machinery, other transport related circumstance, struck by or collision with object, struck by or collision with person, submersion or drowning and unspecified external cause.

ISS: Injury severity score.

GCS: Glasgow Coma Score.

ICU: intensive care unit.

### Follow-up and comparison of responders and non-responders

**Figure 1:**
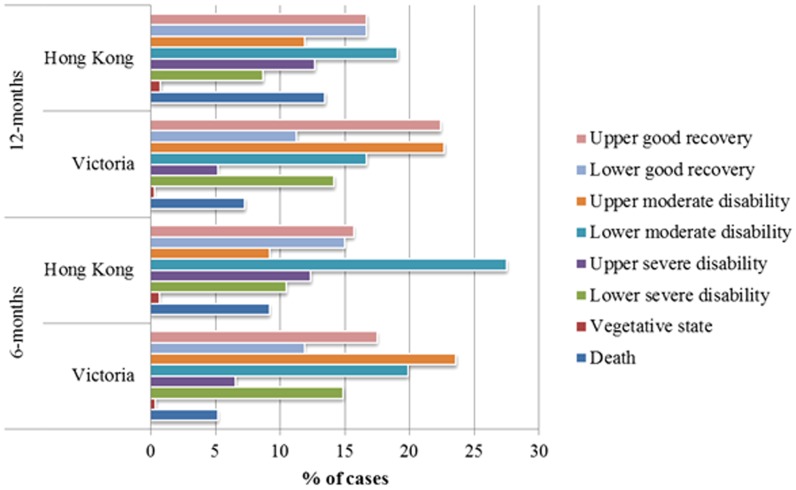
Distribution of GOS-E scores. This figure shows the distribution in GOSE scores between Hong Kong and Victoria.

Including the deaths in-hospital, there was a known GOS-E score at 6-months for 83.4% (n = 1664), and 85.8% (n = 1678) at 12-months for VSTR cases. For cases from Hong Kong, the follow-up rate was 72.4% (n = 189) at 6-months, and 62.1% (n = 162) at 12-months. There were 261 (11.1%) cases lost to follow-up (no known outcome at 6 or 12-months post-injury). There was a bias in non-responders towards being younger, healthier, less severely injured, and penetrating trauma cases (Table 2). Table 2 is pooled data looking at those successfully followed up versus those lost to follow up. The distribution of GOS-E scores at each time point is shown in Figure 1.

**Table 2:** Characteristics of responders and non-responders at follow-up.

Descriptor		Responders (n = 1955)	Non-responders (n = 261)	p-value
Age	Mean (SD)	52.9 (22.5)	46.8 (21.0)	<0.0001
Sex	n (%)			
	Male	1380 (70.6)	185 (70.9)	0.92
	Female	575 (29.4)	76 (29.1)	
Comorbid status	n (%)			
	Healthy	1122 (57.6)	168 (64.6)	0.03
	Pre-existing condition	827 (42.4)	92 (35.4)	
Trauma type	n (%)			
	Blunt	1860 (95.1)	240 (91.9)	0.001
	Penetrating	57 (2.9)	19 (7.3)	
	Burn	38 (1.9)	2 (0.8)	
Mechanism	n (%)			
	Fall	777 (39.7)	86 (33.0)	0.001
	Motor vehicle	409 (20.9)	42 (16.1)	
	Pedestrian	143 (7.3)	25 (9.6)	
	Motorcycle	213 (10.9)	24 (9.2)	
	Pedal cyclist	92 (4.7)	22 (8.4)	
	Other	321 (16.4)	62 (23.8)	
ISS	Median (IQR)	19 (16–26)	17 (14–25)	<0.0001
GCS	n (%)			
	13–15	1505 (80.3)	219 (84.9)	0.001
	9–12	161 (8.6)	29 (11.2)	
	3–8	209 (11.2)	10 (3.9)	
ICU stay	n (%)			
	Yes	802 (41.1)	57 (33.5)	0.018
	No	1149 (58.9)	173 (66.5)	
Hospital length of stay	Median (IQR) days	8.3 (4.1–16.6)	7.2 (3.8–13.4)	0.05

### Prediction of 6-month GOS-E

The unadjusted odds of a better functional outcome at 6-months was 0.67 (95% CI: 0.51, 0.87) for Hong Kong cases compared to major trauma cases from Victoria. Adjusting for age, sex, ISS, comorbid status, mechanism of injury and GCS group, the odds of a better functional outcome for Hong Kong patients relative to Victorian patients was 0.88 (95% CI: 0.66, 1.17) (Table 3).

**Table 3:** Multivariate analysis of association between trauma setting and GOSE at 6-months.

Covariate		Adjusted odds ratio (95% CI)
Setting	Victoria (reference)	1
	Hong Kong	0.88 (0.66, 1.17)
Sex	Male (reference)	1
	Female	0.87 (0.72, 1.06)
Age (years)		0.972 (0.967, 0.976)
ISS		0.95 (0.90, 1.01)
Mechanism	Fall (reference)	1
	Motor vehicle	1.23 (0.96, 1.58)
	Motorcycle	1.37 (1.01, 1.84)
	Pedal cyclist	2.03 (1.33, 3.10)
	Pedestrian	1.20 (0.85, 1.69)
	Other	1.17 (0.89, 1.53)
GCS	13–15 (reference)	1
	9–12	0.44 (0.32, 0.59)
	3–8	0.12 (0.09, 0.17)
Comorbid status	Healthy	1
	Pre-existing condition	0.90 (0.75, 1.07)

ISS: Injury severity score.

GCS: Glasgow Coma Score.

ICU: intensive care unit.

### Prediction of 12-month GOS-E

The unadjusted odds of a better functional outcome at 12-months was 0.58 (95% CI: 0.43, 0.77) for Hong Kong cases compared to major trauma cases from Victoria. Adjusting for age, sex, ISS, comorbid status, mechanism of injury and GCS group, the odds of a better functional outcome for Hong Kong patients relative to Victorian patients was 0.83 (95% CI: 0.60, 1.12) (Table 4).

**Table 4:** Multivariate analysis of association between trauma setting and GOSE at 12-months after injury.

Covariate		Adjusted odds ratio (95% CI)
Setting	Victoria (reference)	1
	Hong Kong	0.83 (0.60, 1.12)
Sex	Male (reference)	1
	Female	0.80 (0.66, 0.98)
Age (years)		0.967 (0.963, 0.972)
ISS		0.96 (0.95, 0.97)
Mechanism	Fall (reference)	1
	Motor vehicle	1.11 (0.87, 1.42)
	Motorcycle	1.49 (1.10, 2.01)
	Pedal cyclist	2.97 (1.89, 4.65)
	Pedestrian	1.11 (0.79, 1.56)
	Other	1.25 (0.95, 1.64)
GCS	13–15 (reference)	1
	9–12	0.51 (0.38, 0.70)
	3–8	0.13 (0.10, 0.18)
Comorbid status	Healthy	1
	Pre-existing condition	0.87 (0.73, 1.04)

ISS: Injury severity score.

GCS: Glasgow Coma Score.

### Comparison of 6- and 12-month SF-12 scores

Valid SF-12 scores were recorded for 855 VSTR cases at 6-months and 861 cases at 12-months. The SF-12 scores were available for 102 Hong Kong cases at 6-months and 76 cases at 12-months. Table 5 shows the mean and standard deviation of the physical (PCS-12) and mental (MCS-12) summary scores of the SF-12 at 6 and 12-months post-injury. There was no difference between the mean PCS-12 scores for Hong Kong patients and Victorian patients at 6-months (mean difference 1.1 (95% CI: −1.3, 3.4) points, p = 0.39) and at 12-months (mean difference −0.3 (95% CI: −3.1, 2.5) points, p = 0.82). There was no difference in the MCS-12 scores between the settings at 6-months (mean difference 1.3 (95% CI: −1.1, 3.6) points, p = 0.29) or 12-months (mean difference 1.9 (95% CI: −0.7, 4.6) points, p = 0.15).

**Table 5:** Health-related quality of life of Hong Kong and Victorian major trauma patients (SF-12 summary scores).

		Hong Kong	VSTR
SF-12 summary score		Mean (SD)	Mean (SD)
**PCS-12**	6-months	42.7 (9.8)	41.6 (11.8)
	12-months	42.2 (11.0)	42.6 (12.0)
**MCS-12**	6-months	51.8 (12.4)	50.6 (11.4)
	12-months	52.2 (10.9)	50.3 (11.2)

SF: Short-Form.

PCS: Physical Component Summary.

MCS: Mental Component Summary.

Adjusting for age, gender, ISS, GCS, mechanism of injury and the presence of comorbid conditions, Hong Kong patients demonstrated comparable mean PCS-12 scores at 6-months (adjusted mean difference: 1.2, 95% CI: −1.2, −3.6) and 12-months (adjusted mean difference: 0.4, 95% CI: −3.2, 2.4) compared to Victorian major trauma patients. Keeping age, gender, ISS, GCS, mechanism of injury and the presence of comorbidities steady, there was no difference in the MCS-12 scores of Hong Kong patients compared to Victorian patients at 6-months (adjusted mean difference: 0.4, 95% CI: −2.1, 2.8) or 12-months (adjusted mean difference: 1.8, 95% CI: −0.8, 4.5).

## Discussion

This is the first study to compare six- and 12-month functional outcome of patients with serious trauma across two regions in different countries. After adjusting for known predictors, there was no difference in the six- and 12-month odds ratio for a good outcome (high GOSE) between the two countries although for a given injury there was a trend towards a more favourable outcome in Australia than Hong Kong.

There are a number of reasons why Victoria was chosen for benchmarking. Firstly, there is a long history of research between our two centres. We have already published benchmarking studies on post-trauma mortality between our two centres. It naturally follows that if possible we should go on to compare other outcomes. Secondly, we have collected data on post-trauma health outcome using similar tools, which allow meaningful comparison. There are few centres around the world that use comparable methods of data collection. Thirdly, Victoria is a centre of excellence in trauma research.

Survivors of trauma often experience late sequelae that have a major impact on almost all aspects of their everyday life [7]–[9]. The majority of severely injured patients survive their injury but the disruption to their lives and cost to society are substantial. Patient-centred, health-related outcomes are increasingly recognized as an important benchmark of the quality of care received yet there is a little information at a system level. Meaningful comparisons between different regional centres enable healthcare providers to assess the current standard of care, and whether there are clear areas for improvement.

This study was not designed to identify differences in the quality of the prehospital care, acute in-hospital management or rehabilitation between the two centres. There are some obvious differences between the two regions, which are important to consider.

Firstly, a greater percentage of Gross Domestic Product is spent on health care in Australia than that in Hong Kong (9% v 5.9%) [34]. Secondly, paramedics in Victoria undertake a three-year university course culminating in a bachelors degree level training, whilst in Hong Kong many paramedics have only a secondary school education, and less than 25% have any form of university degree. Thirdly, emergency department staffing levels in Victoria are very different to those in Hong Kong. For example, the ratio of emergency department medical staff to new patient attendances in the Alfred Hospital, Victoria is approximately 1∶1000 which compares with that in Prince of Wales Hospital, Hong Kong currently set at 1∶7000. Such staffing levels are likely to affect the levels of acute hospital and trauma care and outcomes.

Currently there is little or no data available on the quantity or quality of rehabilitation, the success of pain relieving strategies, or of the psychological perceptions of trauma which would allow any meaningful comparison between Hong Kong and Victoria but it is likely that these are major factors that affect recovery after injury.

Should we have used regional or US weights for the SF-12 comparisons between HK and Australia? Weighting is meant to normalise data to a region so that regional population bias is minimised. However, using different weights may theoretically produce different effects based on weighting rather than the trauma. Therefore whether or not to use different weights is a complex issue for which there is no agreed consensus. We also note that although the ethnic mix between the US, HK and Australia is different, nevertheless the population norms for MCS and PCS are not that different. The US and Australian normative weights are almost exactly the same i.e. <1%. In HK the variation in mean PCS and MCS from the US norm is <±3%. As can be seen by the data, the effects of major trauma on MCS and PCS are huge such that a possible error of <3% is acceptable. Our final decision was to use US weights which neither favoured HK nor Australia.

The cohort from Hong Kong are Chinese rather than Caucasian, are more likely to have pre-injury co-morbidity, more falls, less car occupants, a higher ISS, lower GCS, and are more likely to be admitted to ICU, and to stay longer in the acute hospital setting. After adjusting for these factors, the quality of life and functional outcome at 6 and 12 months in major trauma patients between Victoria and Hong Kong were comparable. We looked at quality of life for survivors to hospital discharge as it is expected that initial hospital treatments in a trauma system will affect survival. The fact that survivors were comparable in outcomes between systems with differing survival rates is important.

The combined respondents from both settings were more likely to be older, to have pre-injury good health, to have blunt injury rate, a higher ISS, lower GCS, and to have stayed in the acute hospital longer.

### Limitations

The response rate was higher in Victoria than Hong Kong. In some cases there are missing data and difficulty with follow up. This is a feature of all such studies but is a greater problem in Hong Kong (62%) than in Victoria (86%). The disparity in sample size between HK and Australia strains the statistical analysis but is a limitation that needs to be accepted at this stage of study. Comparisons between top level centres is important for benchmarking and quality evaluation, and is well supported by healthcare providers and managers, and supported with funding by government who have a major interest in evaluating systems, quality of care and outcomes. The best methods for such comparisons for long-term morbidity and quality of life have never been defined, and as such this study is explorative rather than definitive. It highlights issues that need to be addressed in future, and provides insights into appropriate sample sizes and funding needs for future studies.

Many factors from pre-injury through the whole process of care are likely to impact on long-term outcomes and have not been measured in this study. These include pre-injury education level and socioeconomic status, and multiple post-injury process items such as prehospital care and time from injury to hospital, in hospital surgical, ward and ICU care, physiotherapy and rehabilitation. We are not able to evaluate whether such factors influenced long-term morbidity. The influence of time from injury to emergency room resuscitation is more likely to impact on early mortality rather than on long-term morbidity but we cannot be sure of this. However, the main aim of this study was not to identify major factors that influence outcome but to provide initial data on the overall morbidity. If there were major differences then future studies would be planned to evaluate these.

Repeated measures analysis was not used in this study as the difference in loss to follow-up rates between the two jurisdictions results in substantial imbalance in the data. While some repeated measures models (e.g. mixed and random effects models) may produce robust estimates in the presence of data imbalance, the difference in sample size and follow-up rates between the two settings was too large for these models to converge with meaningful estimates of the association. We considered that the alternative, a regression model for each time point post-injury, while not ideal, was the best option for this study.

There was no available pre-existing data on which to determine an a priori power calculation for quality of life follow up as this is the first study of its type. In HK the 12-month PCS is 51.80 (SD 12.4), and in Australia it is 50.60 (SD 11.4), a difference of 3.04. There is an eight-fold difference in sample size between VSTR and HK. A 2–3 point difference in mean PCS or MCS is considered an important difference when comparing groups and populations.

Using a two-tailed test, and a sample ratio of 8∶1, 2007 patients are required in order to have a 80% chance of detecting, as significant at the 5% level, to detect a 2-point difference in the mean PCS score assuming a pre-existing mean of 43.6 and a SD of 11.8, that is 1784 patients in the control group and 223 patients in the comparator group [35].

These are important conclusions from this study, which future researchers and funding bodies will have to consider. It is important to note that VSTR is awarded 10 times the amount of fund to complete such research as is awarded in Hong Kong. The sample sizes from both VSTR and Hong Kong are sufficient for a well-powered study. However, the high non-response rate from Hong Kong means that the current study is underpowered. Further, although the absolute mean PCS between Hong Kong and Australia was similar, the fact that Australia has a lower population norm than Hong Kong suggests that the adjusted outcomes in Australia are better than Hong Kong.

### Conclusion

This is the first attempt to compare quality of life outcomes between Australia and Hong Kong. The unadjusted analyses showed better outcomes for Victorian cases compared to Hong Kong but after adjusting for key confounders, there was no difference in 6-month or 12-month functional outcomes between the jurisdictions.
